# Risk Estimation of Late Rectal Toxicity Using a Convolutional Neural Network-based Dose Prediction in Prostate Cancer Radiation Therapy

**DOI:** 10.1016/j.adro.2025.101739

**Published:** 2025-02-15

**Authors:** Seiya Takano, Natsuo Tomita, Taiki Takaoka, Machiko Ukai, Akane Matsuura, Masanosuke Oguri, Nozomi Kita, Akira Torii, Masanari Niwa, Dai Okazaki, Takahiro Yasui, Akio Hiwatashi

**Affiliations:** a Department of Radiology, Nagoya City University Graduate School of Medical Sciences, 1 Kawasumi, Mizuho-cho, Mizuho-ku, Nagoya, Aichi, Japan; b Department of Urology, Nagoya City University Graduate School of Medical Sciences, 1 Kawasumi, Mizuho-cho, Mizuho-ku, Nagoya, Aichi, Japan

## Abstract

**Purpose:**

The present study investigated the feasibility of our automatic plan generation model based on a convolutional neural network (CNN) to estimate the baseline risk of grade ≥2 late rectal bleeding (G2-LRB) in volumetric modulated arc therapy for prostate cancer.

**Methods and Materials:**

We built the 2-dimensional U-net model to predict dose distributions using the planning computed tomography and organs at risk masks as inputs. Seventy-five volumetric modulated arc therapy plans of prostate cancer, which were delivered at 74.8 Gy in 34 fractions with a uniform planning goal, were included: 60 for training and 5-fold cross-validation, and the remaining 15 for testing. Isodose volume dice similarity coefficient, dose-volume histogram, and normal tissue complication probability (NTCP) metrics between planned and CNN-predicted dose distributions were calculated. The primary endpoint was the goodness-of-fit, expressed as a coefficient of determination (*R*^2^) value, in predicting the percentage of G2-LRB-Lyman-Kutcher-Burman-NTCP.

**Results:**

In 15 test cases, 2-dimensional U-net predicted dose distributions with a mean isodose volume dice similarity coefficient value of 0.90 within the high-dose region (doses ≥ 50 Gy). Rectum V_50Gy_, V_60Gy_, and V_70Gy_ were accurately predicted (*R*^2^ = 0.73, 0.82, and 0.87, respectively). Strong correlations were observed between planned and predicted G2-LRB-Lyman-Kutcher-Burman-NTCP (*R*^2^ = 0.80, *P* < .001), with a small percent mean absolute error (mean ± 1 standard deviation, 1.24% ± 1.42%).

**Conclusions:**

A risk estimation of LRB using CNN-based automatic plan generation from anatomic information was feasible. These results will contribute to the development of a decision support system that identifies priority cases for preradiation therapy interventions, such as hydrogel spacer implantation.

## Introduction

Late rectal bleeding (LRB) is one of the most important sequelae after external radiation therapy (RT) for prostate cancer, despite the use of volumetric modulated arc therapy (VMAT).^1^^,^^2^ The risk of late rectal toxicity is dependent on the proximity between the prostate and the anterior rectal wall, and thus, one solution is to implant an absorbable polyethylene glycol hydrogel spacer between the Denonvilliers fascia and rectal wall before RT.^3^^,^^4^ Hydrogel spacer implantation is associated with a 77% lower risk of late grade ≥ 2 rectal toxicities by reducing the high-dose area of the rectum, such as V_70Gy_.^5^ However, using a hydrogel spacer in all cases may be inappropriate and not cost-effective because of its invasiveness, potential complications, and low rate of LRB after RT without one.^1^^,^^2^^,^^6^, ^7^, ^8^ Although previous studies attempted to analyze the harm-benefit ratio of hydrogel spacer implantation based on a rectal normal tissue complication probability (NTCP) model,^7^^,^^9^ it requires time-consuming treatment planning for decision-making. In this context, we propose a method to estimate the baseline risk of LRB using the automatic plan generation model based on a convolutional neural network (CNN) from anatomic information. The proposed method may serve as a decision support tool to identify priority cases for hydrogel spacer implantation.

CNN has recently emerged as a promising approach for the generation of 3-dimensional (3D) dose distributions from delineations on planning computed tomography (CT) scans.^10^, ^11^, ^12^, ^13^, ^14^, ^15^, ^16^ CNN-based dose predictions have been reported to outperform conventional machine learning approaches, such as the overlap volume histogram (OVH) method for predicting the lowest achievable dose-volume histogram (DVH).^13^^,^^16^ However, it remains unknown whether CNN-predicted 3D dose distributions provide an accurate estimate of NTCP values, which take into account the biological effects of nonuniform dose distributions. Therefore, the present study investigated the feasibility of our CNN-based automatic plan generation model to estimate the baseline risk of grade ≥ 2 late rectal bleeding (G2-LRB) in prostate cancer VMAT from anatomic information. We focused on G2-LRB because the primary goal of hydrogel spacer implantation is to prevent LRB following RT, and symptoms requiring medical intervention have greater predictive value.^4^^,^^5^

## Methods and Materials

### Patient selection

We collected the treatment plans of 84 consecutive patients with clinical stage T1-3N0M0^17^ prostate cancer who received definitive VMAT at our institution between 2015 and 2018. Of these 84 patients, 7 low-risk patients according to the D'Amico stratification were also excluded because of a lower prescribed dose: 72.6 Gy in 33 fractions for low-risk patients and 74.8 Gy in 34 fractions for intermediate- and high-risk patients. In addition, one patient with missing rectum delineation because of a history of abdominoperineal resection of the rectum and another with treatment planning based on magnetic resonance imaging alone were excluded. This yielded an entire data set comprising 75 patients along with their respective treatment plans. No patients underwent hydrogel spacer implantation. The present study was conducted in compliance with the Declaration of Helsinki and its later amendments and was approved by the Institutional Review Board. Because of the retrospective nature of this analysis, the Institutional Review Board waived the need for informed consent as part of the study approval in compliance with the regulations in our country regarding medical research involving human subjects. The research content was disclosed in the form of an opt-out on the website.

### Treatment procedures

Patients were instructed to void the bladder 1 hour before the planning CT scan and each treatment to achieve adequate bladder filling and to attempt a bowel movement before arrival. If rectal gas or stool was evident on the planning CT scan or daily cone-beam CT scan, patients were instructed to evacuate, and rectal gas was removed using a catheter if necessary. Planning CT scan was performed using GE Optima CT580W (GE Healthcare Technologies, Inc). All treatments were planned with the collapsed cone convolution algorithm at a resolution of 2.0 × 2.0 × 2.0 mm using the RayStation version 4.5 treatment planning system (RaySearch Laboratories). VMAT was performed on TrueBeam (Varian Medical Systems, Inc) with 1 or 2 coplanar full arcs with 10-MV photon beams. The clinical target volume and planning target volume (PTV) were delineated as previously described.^1^ All structure regions of interest (ROIs) were contoured in the range of PTV ± 1 cm craniocaudally. The total dose of 74.8 Gy in 34 fractions was prescribed to 50% of the PTV. Treatment plans were optimized to achieve the following planning goals for the PTV: at least 95% of the prescribed dose was delivered to 95% of the PTV, the maximum dose was limited to 110% of the prescribed dose, the mean dose (D_mean_) was maintained within 99% to 103% of the prescribed dose, and V_67.3Gy_ ≥ 96%. For the rectum, dose constraints were V_75.1Gy_ ≤ 0%, V_57.7Gy_ ≤ 18%, and V_38.5Gy_ ≤ 35% (Table E1). Dose constraints for the bladder, bowel, and femoral heads are also provided in Table E1.

### Preprocessing

To prevent graphics processing unit memory overflow, the matrix size of each CT, structure ROI, and dose distribution (image matrix, 512 × 512; maximum slice number, 81 to 156; pixel spacing, 0.98 mm; and slice thickness, 2.5 mm) was cropped to 400 × 400 pixels and finally reduced to 128 × 128 pixels at a resolution of 3.06 × 3.06 mm^2^. Additionally, up to 80 consecutive slices that sufficiently encompassed the region within 1 cm cranially and caudally from the PTV were selected as input images. All dose values for both training and testing data sets were rescaled such that 95% of the PTV received 95% of the prescribed dose. This rescaling was applied to minimize the effect of outliers in target coverage for the training data set and to enable predictions based on reasonable target coverage estimates from the optimization protocol for the testing data set. For the training data set, the dose values were subsequently normalized by the global maximum dose across the entire training data set to sufficiently incorporate data from high-dose regions of the rectum into the model.

### Training and validation

**Figure 1:**
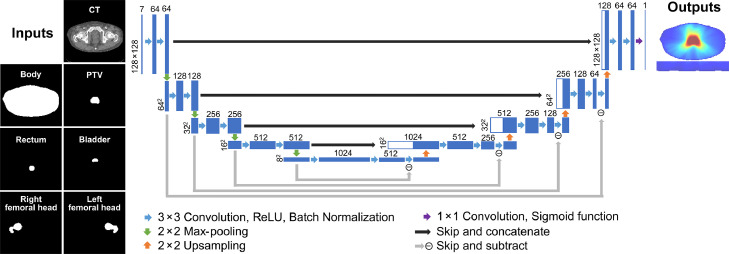
Schematic diagram of the 2-dimensional (2D) U-net architecture for dose predictions in prostate volumetric modulated arc therapy. The number above each box represents the number of features for each map, while the numbers at the left of each box represent the size of each 2D feature. *Abbreviations*: ReLU = rectified linear unit.

We developed a 2-dimensional (2D) U-net model in Python 3.8.2 using Keras with Tensorflow version 2.11.0 as the backend.^10^^,^^16^^,^^18^ Figure 1 shows the architecture of the 2D U-net model, which consists of the input, encoding block, decoding block, skip connection and output. The input consisted of 7 channels of 128 × 128 matrices containing the CT, 4 organs at risk (OARs; rectum, bladder, left femoral head, and right femoral head), PTV, and body. To combine local and global feature maps, the outputs of each encoding and decoding block were concatenated with the skip connection. Residual connections were added between corresponding convolutional layers in the encoding and decoding blocks to enhance model training.^19^ To avoid overfitting, a dropout layer (dropout rate of 0.1) was applied after every 2 batch normalization layers. The output was the predicted dose distribution with a single channel of 128×128 matrices.

Seventy-five patients were randomly split into subsets of 60 patients for training and 15 for testing. The size of input images was 4784 slices × 7 channels for training and 1200 slices × 7 channels for testing, respectively. We used a 5-fold cross-validation by randomly dividing the training data set into 5 validation subsets of 12 patients for each fold. The final model was built by averaging the 5 U-net models. The predictive performance of the final model was evaluated using the 15 test patients. The loss function was defined as the mean squared error between the planned and predicted doses. The adaptive moment estimation was applied as an optimizer with a learning rate of 1.0 × 10^−3^, default parameters (*β_1_* = 0.9, *β_2_* = 0.999, *weight decay* = 0), and a mini-batch size of 15 images. Each cross-validation model was trained for 250 epochs using an NVIDIA Tesla V100 graphics processing unit with 16 GB of memory.

### Model performance and DVH analysis

To quantify the similarity between planned and predicted 3D dose distributions, we calculated the isodose volume dice similarity coefficient (iDSC) given by

(1) $$\text{iDSC}\;\left(d\right)=\;\frac{2\left(V_{planned}\left(d\right)\cap V_{predicted}\left(d\right)\right)}{V_{planned}\left(d\right)\cup V_{predicted}\left(d\right)},$$

where *V* refers to the isodose volume of the dose *d* (Gy) ranging from 0 to the prescribed dose.^20^ The mean iDSC_≥0Gy_ and iDSC_≥50Gy_ were defined as the averages of iDSC values for doses ≥ 0 Gy and ≥ 50 Gy, respectively. The iDSC_≥0Gy_ was used to assess the overall agreement of dose distributions, while the iDSC_≥50Gy_ focused on evaluation within the high-dose region. Additionally, the following DVH metrics were calculated: D_98%_, D_95%_, D_50%_, D_2%_, D_mean_, homogeneity index (HI = 100·[D_2%_− D_98%_]/[ D_50%_]), and R50% = 100·V_50%isodose_ /V_PTV_ for the PTV; V_70Gy_, V_60Gy_, V_50Gy_, V_40Gy_, D_2%_, and D_mean_ for the rectum and bladder; and D_2%_ and D_mean_ for the right and left femoral head. D*_x_*_%_ is the minimum dose delivered to *x*% the structure. V*_xGy_* is the percentage of the organ receiving at least *x* Gy. The percent mean absolute errors (%MAEs) of the DVH and NTCP metrics between the planned and predicted dose distributions were calculated as follows: for Gy-unit metrics (eg, D_2%_, D_mean_), %MAE was defined as the mean of 100·|Predicted (Gy) − Planned (Gy)|/Prescribed dose (Gy); for %unit metrics (eg, V_70Gy_, NTCP), %MAE was defined as the mean of |Predicted (%) − Planned (%)|.

### NTCP analysis

The NTCP values for grade ≥ 1 LRB (G1-LRB-NTCP) and grade ≥ 2 LRB (G2-LRB-NTCP) were calculated based on planned and CNN-predicted 3D dose distributions. These NTCP values were regarded as surrogate endpoints for LRB after prostate RT. G2-LRB-NTCP values were calculated using the Lyman-Kutcher-Burman (LKB) and relative seriality (RS) models. The LKB model was defined as follows:

(2) $$\text{NTCP}\;=\;\frac{1}{\sqrt{2\pi }}\int_{-\infty }^{t}\text{exp}\left(-\frac{x^{2}}{2}\right)dx$$

(3) $$t\;=\;\frac{D_{eff}-TD_{50}}{mTD_{50}},$$

where *TD_50_* represents the tolerance dose of 50% toxicity; *m* represents the steepness of the dose-response curve; and *D_eff_* is the effective dose, given by

(4) $$D_{eff}\;=\;{\left(\sum_{i=1}^{M}{\left(EQD2_{i}\right)}^{\frac{1}{n}}\cdot v_{i}\right)}^{n},$$

where *n* represents the volume effect of the organ with a value toward 0 being more serial; *v_i_* represents the relative volume of the organ at the dose bin *i; M* represents the total number of dose bins; and EQD2*_i_* represents an equivalent dose in 2-Gy fractions at the dose bin *i*. EQD2*_i_* was calculated according to the linear-quadratic formula with α/β = 3.0 Gy.^21^ The following parameters were used: *n* = 0.23, *m* = 0.37, *TD_50_* = 57.3 Gy for G1-LRB-NTCP; *n* = 0.19, *m* = 0.32, *TD_50_* = 75.8 Gy for G2-LRB-NTCP; *n* = 0.27, *m* = 0.56, *TD_50_* = 55.7 Gy for G1 stool frequency; *n* = 0.31, *m* = 0.36, *TD_50_* = 75.8 Gy for G2 stool frequency; *n* = 0.17, *m* = 0.49, *TD_50_* = 142.6 Gy for G1 bowel pain; *n* = 0.24, *m* = 0.32, *TD_50_* = 79.1 Gy for G1 sphincter control; and *n* = 0.32, *m* = 0.25, *TD_50_* = 84.4 Gy for G1 stricture or ulcer. These parameters were previously validated by Brand et al*.*^22^ According to the grading system employed in their studies, grade 1 indicates toxicity not requiring an intervention, while grade ≥ 2 indicates toxicity requiring any intervention.

The RS model was defined as follows:

(5) $$\text{NTCP}\;=\;{\left\{1-\prod_{i=1}^{M}{\left[1-P{\left(D_{i}\right)}^{s}\right]}^{v_{i}}\right\}}^{\frac{1}{s}},$$

where *s* is the parameter of seriality; *v_i_* represents the relative volume of the organ; *M* represents the total number of dose bins; *D_i_* represents an EQD2 at the dose bin *i*; and *P(*$D_{i}$*)* represents the Poisson dose-response relationship, given by

(6) $$P\left(D\right)\;=\;2^{-\exp\left\{e\gamma \cdot \left(1-\frac{D}{D_{50}}\right)\right\}},$$

where *D_50_* represents the tolerance dose of 50% toxicity and *γ* represents the slope of the response curve at *D_50_*. The following parameters were used to calculate G2-LRB-NTCP: *D_50_* = 83.6 Gy, *γ* = 1.42, *s* = 0.50, and α/β = 3.0 Gy.^23^ In this model, grade 1 indicates LRB occurring up to twice a week, while grade 2 indicates LRB occurring more than twice a week, as described by Cicchetti et al.^24^

### Statistical analysis

The primary endpoint was the goodness-of-fit of the linear regression model predicting G2-LRB-NTCP in the LKB model (G2-LRB-LKB-NTCP). The goodness-of-fit was evaluated using the coefficient of determination (*R*^2^) values, ranging from 0 to 1, with a larger value indicating a better fit. For sensitivity analysis, the goodness-of-fit was also evaluated for G2-LRB-NTCP using the RS model (G2-LRB-RS-NTCP). The *P* value was calculated with the null hypothesis that the regression coefficient equals zero using the Wald test with the *t*-distribution of the test statistic. With a sample size of 15, this study had 80% power at a significance level of α value of 0.05 for detecting an *R*^2^ value of 0.35. The goodness-of-fit was also evaluated for the other DVH metrics. All statistical analyses were conducted using SciPy version 1.7.3. The threshold for significance was *P* < .05.

## Results

**Figure 2:**
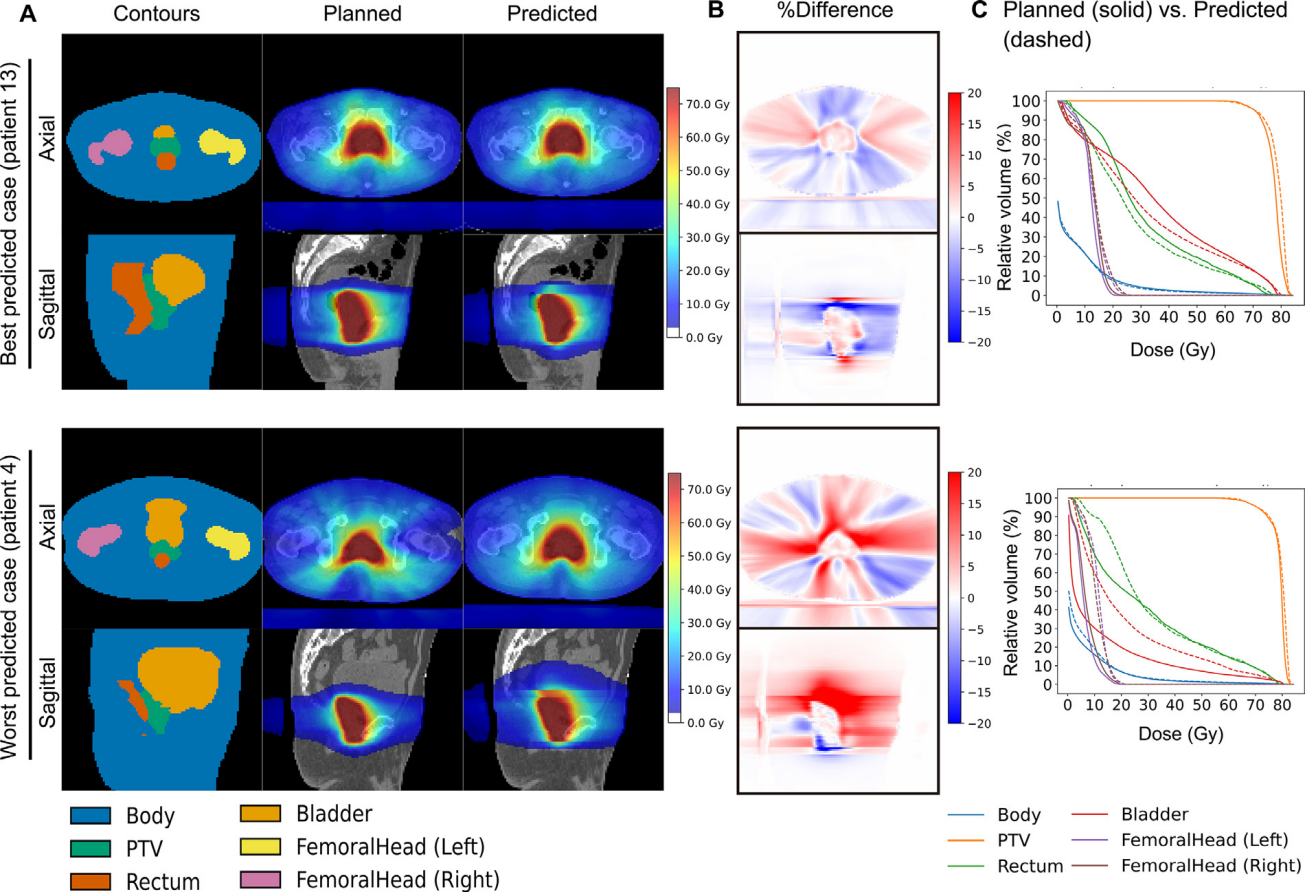
Best and worst examples of dose predictions with mean iDSC_≥0Gy_ values of 0.91 and 0.82, respectively. Each column shows: (A) contours and planned and predicted dose distributions; (B) percent dose difference maps (predicted − planned); and (C) comparisons between planned (solid line) and predicted (dashed line) dose-volume histograms. *Abbreviations*: iDSC = isodose volume dice similarity coefficient; PTV = planning target volume.

**Figure 3:**
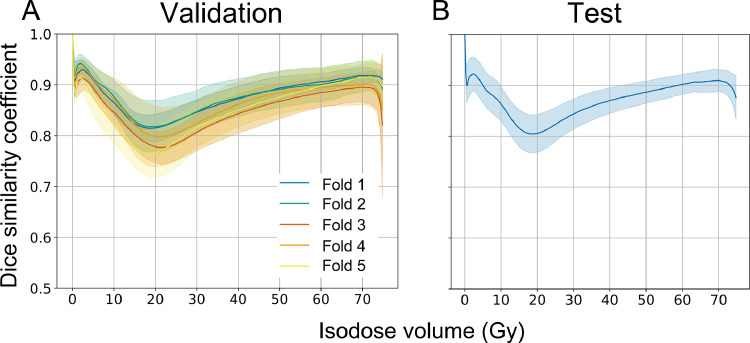
Plots of isodose volume dice similarity coefficient in (A) the 5-fold cross-validation (n = 12 per fold) and (B) testing (n = 15). Data represent means ± 1 SD.

Figure E1 shows the 2 representative dose distributions in the 60 training cases. The G2-LRB-LKB-NTCP values in the training cases ranged from 9.69% to 15.01% (Fig. E1). Model training took approximately 7 hours, while the prediction of 3D dose distributions in 15 test cases required approximately 20 seconds. Training and validation loss curves from one of the folds are shown in Fig. E2. The average losses ± 1 SD of training and cross-validation were (2.92 ± 0.42) × 10^−5^ and (4.83 ± 0.99) × 10^−4^, respectively. Figure 2 shows representative images for the best-predicted (patient 13, mean iDSC_≥0Gy_ = 0.91) and worst-predicted cases (patient 4, mean iDSC_≥0Gy_ = 0.82). Predicted 3D dose distributions showed concave-shaped dose fall-offs toward the rectum and intermediate doses (30-50 Gy) spreading toward the bladder, similar to planned dose distributions (Fig. 2A). The predicted dose distribution lacked continuity in some axial planes that did not encompass the PTV (Fig. 2A). The CNN-predicted dose distribution and DVH generally aligned with those achieved clinically (Fig. 2B, C). Figure 3 shows the plots of iDSC_≥0Gy_ in cross-validation and test cases. The iDSC_≥0Gy_ for test cases ranged from 0.80 to 0.92. Mean iDSC_≥0Gy_ values were 0.87 ± 0.01 for the 5-fold cross-validation and 0.87 for testing. Mean iDSC_≥50Gy_ values were 0.90 ± 0.01 for the 5-fold cross-validation and 0.90 for testing.

**Figure 4:**
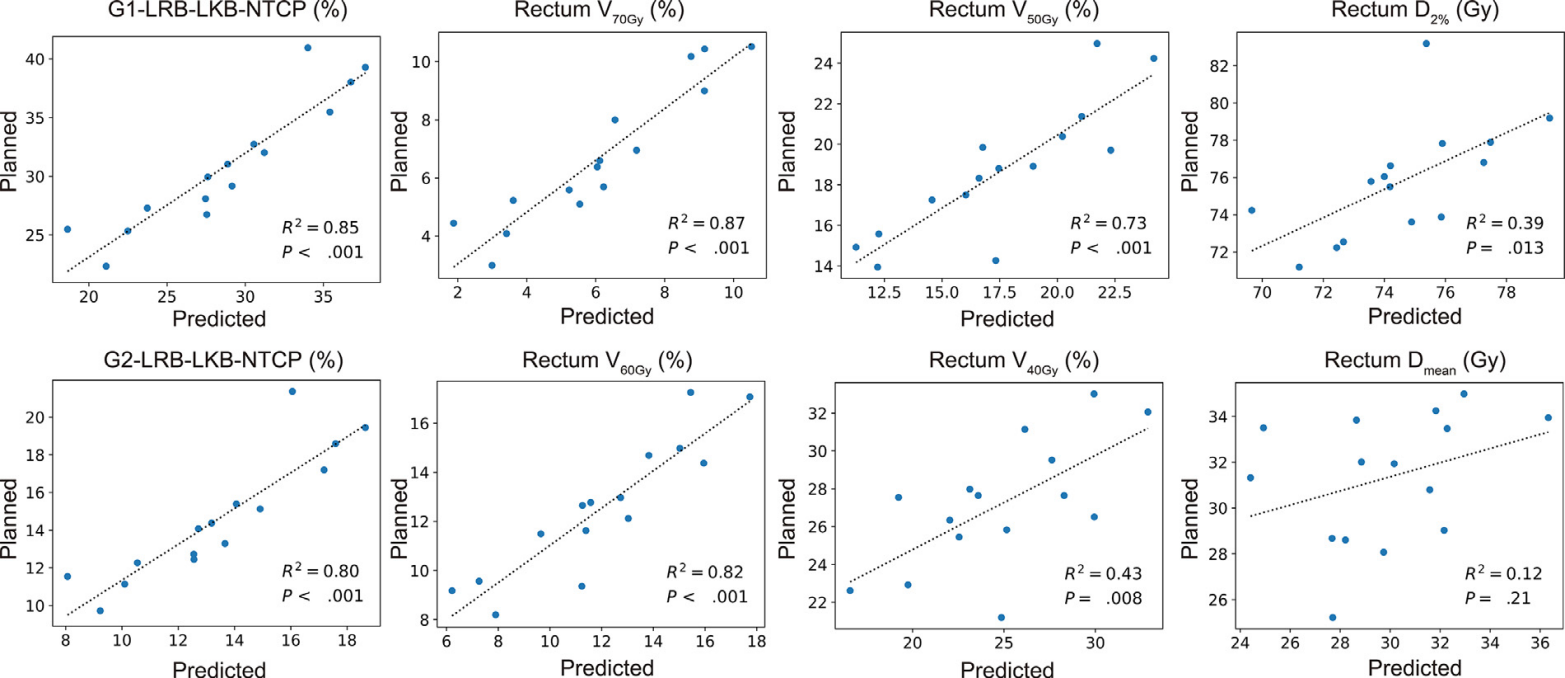
Scatter plots of the linear regression analysis between planned and predicted NTCP and DVH metrics. *Abbreviations*: DVH = dose-volume histogram; G1 = grade ≥ 1; G2 = grade ≥ 2. LRB = late rectal bleeding; LKB = Lyman-Kutcher-Burman model; NTCP = normal tissue complication probability.

Table 1 shows %MAEs ± 1 SD between planned and predicted metrics for the 15 test cases. %MAEs were 2.22 ± 2.15% for G1-LRB-LKB-NTCP, 1.24 ± 1.42% for G2-LRB-LKB-NTCP, and 0.23 ± 0.15% for G2-LRB-RS-NTCP. The ranges of percent absolute errors were 0.01–6.96% for G1-LRB-LKB-NTCP and 0.02–5.31% for G2-LRB-LKB-NTCP. %MAEs were within 5.00% for PTV D_98%_, D_95%_, D_50%_, and D_2%_; within 2.00% for rectum V_50Gy_–V_70Gy_; and within 6.00% for bladder V_50Gy_–V_70Gy_. The linear regression analysis indicated strong correlations between planned and predicted rectal NTCP values (*R*^2^ = 0.85, *P* < .001 for G1-LRB-LKB-NTCP; *R*^2^ = 0.80, *P* < .001 for G2-LRB-LKB-NTCP, Fig. 4). Strong correlations were also observed in the G2-LRB-RS-NTCP (*R*^2^ = 0.93, *P* < .001, Fig. E3) and other endpoints, including G1 stool frequency (*R*^2^ = 0.86), G2 stool frequency (*R*^2^ = 0.87), G1 bowel pain (*R*^2^ = 0.78), G1 sphincter control (*R*^2^ = 0.85), and G1 stricture or ulcer (*R*^2^ = 0.89, Fig. E3). A rectal volume receiving higher doses was more likely to yield significantly higher *R*^2^ values (*R*^2^ = 0.43, 0.73, 0.82, and 0.87 for rectum V_40Gy_, V_50Gy_, V_60Gy_, and V_70Gy_, respectively). The *R*^2^ of rectum D_mean_ was 0.12 with no significance (*P* = .21).

**Table 1:** Comparison of DVH and NTCP metrics between planned and predicted dose distributions for 15 test cases

Structure	Metrics	Planned	Predicted	%MAE	*R*^2^	*P* value
PTV						
	D_98%_ (Gy)	66.91 ± 1.66	67.42 ± 1.48	1.47 ± 1.53	0.28	.041
	D_95%_ (Gy)	71.06 ± 0.01	71.06 ± 0.02	0.01 ± 0.02	0.00	.98
	D_50%_ (Gy)	79.58 ± 2.52	78.96 ± 2.40	3.16 ± 2.45	0.07	.34
	D_2%_ (Gy)	83.61 ± 3.76	81.50 ± 3.14	4.78 ± 4.20	0.05	.43
	D_mean_ (Gy)	78.76 ± 2.27	78.03 ± 2.22	3.03 ± 2.20	0.06	.39
	HI (%)	20.83 ± 5.75	17.71 ± 4.78	4.73 ± 3.91	0.24	.061
	R50% (%)	430.10 ± 64.90	404.76 ± 68.91	47.80 ± 31.84	0.48	.004
Rectum						
	G1-LRB-LKB-NTCP (%)	30.93 ± 5.50	28.81 ± 5.70	2.22 ± 2.15	0.85	< .001
	G2-LRB-LKB-NTCP (%)	14.58 ± 3.31	13.40 ± 3.12	1.24 ± 1.42	0.80	< .001
	G2-LRB-RS-NTCP (%)	1.41 ± 0.66	1.20 ± 0.63	0.23 ± 0.15	0.93	< .001
	G1 stool frequency (%)	35.23 ± 3.75	33.63 ± 4.19	1.72 ± 1.45	0.86	< .001
	G2 stool frequency (%)	10.54 ± 2.02	9.67 ± 2.20	0.95 ± 0.68	0.87	< .001
	G1 bowel pain (%)	9.74 ± 0.88	9.42 ± 0.85	0.34 ± 0.40	0.78	< .001
	G1 sphincter control (%)	9.64 ± 2.24	8.80 ± 2.21	0.88 ± 0.82	0.85	< .001
	G1 stricture/ulcer (%)	2.06 ± 0.71	1.78 ± 0.71	0.31 ± 0.20	0.89	< .001
						
	V_70Gy_ (%)	6.75 ± 2.40	6.16 ± 2.52	0.77 ± 0.73	0.87	< .001
	V_60Gy_ (%)	12.55 ± 2.78	12.01 ± 3.32	1.22 ± 0.85	0.82	< .001
	V_50Gy_ (%)	18.67 ± 3.30	17.53 ± 3.91	1.90 ± 1.30	0.73	< .001
	V_40Gy_ (%)	27.16 ± 3.39	24.78 ± 4.48	3.52 ± 2.09	0.43	.008
	D_2%_ (Gy)	75.77 ± 3.07	74.54 ± 2.52	2.41 ± 2.77	0.39	.013
	D_mean_ (Gy)	31.31 ± 2.84	29.83 ± 3.14	3.84 ± 3.10	0.12	.21
Bladder						
	V_70Gy_ (%)	13.20 ± 5.50	11.90 ± 4.60	3.19 ± 2.43	0.52	.002
	V_60Gy_ (%)	19.46 ± 7.16	18.18 ± 6.41	4.06 ± 2.36	0.60	< .001
	V_50Gy_ (%)	26.24 ± 9.36	24.76 ± 8.35	5.65 ± 2.63	0.58	.001
	V_40Gy_ (%)	34.85 ± 12.24	33.06 ± 10.86	7.08 ± 3.35	0.60	< .001
	D_2%_ (Gy)	79.13 ± 3.27	77.89 ± 2.59	3.99 ± 3.05	0.06	.36
	D_mean_ (Gy)	31.78 ± 9.29	31.63 ± 7.42	6.50 ± 3.30	0.64	< .001
Right femoral head						
	D_2%_ (Gy)	22.89 ± 5.68	21.73 ± 1.85	4.85 ± 4.77	0.24	.063
	D_mean_ (Gy)	12.19 ± 3.49	12.73 ± 1.04	3.41 ± 2.61	0.15	.15
Left femoral head						
	D_2%_ (Gy)	21.33 ± 4.01	20.11 ± 2.50	4.30 ± 3.99	0.04	.48
	D_mean_ (Gy)	11.40 ± 3.17	11.77 ± 1.27	3.26 ± 2.76	0.02	.60

*Abbreviations*: %MAE = percent mean absolute error; DVH = dose-volume histogram; PTV = planning target volume; HI = homogeneity index; G1 = grade ≥1; G2 = grade ≥2; LRB = late rectal bleeding; LKB = Lyman-Kutcher-Burman; NTCP = normal tissue complication probability; RS = relative seriality.

Data are means ± 1 SD. %MAEs of the DVH and NTCP metrics between the planned and predicted dose distributions were calculated using the following formulas: for Gy-unit metrics (eg, D_2%_, D_mean_), %MAE was defined as the mean of 100·|Predicted (Gy) − Planned (Gy)|/Prescribed dose (Gy); for %-unit metrics (eg, V_70Gy_, NTCP), %MAE was defined as the mean of |Predicted (%) − Planned (%)|. The dose distributions for the testing data set were rescaled such that 95% of the PTV received 95% of the prescribed dose. The coefficient of determination (*R*^2^) and *P* value by a linear regression analysis between planned and predicted metrics are shown. *R*^2^ ranges from 0 to 1, with a larger value indicating a better goodness-of-fit.

## Discussion

This study investigated the feasibility of predicting rectal NTCP in prostate VMAT using CNN-predicted 3D dose distributions based on patient anatomy. In 15 test cases, automatically generated 3D dose distributions provided a good estimation of planned G2-LRB-LKB-NTCP (*R*^2^ = 0.80, *P* < .001; Fig. 4) along with a small prediction error of 1.24% (Table 1). Consistent results were found in the RS model (*R*^2^= 0.93, Fig. E3; %MAE = 0.23%, Table 1). These results indicate the feasibility of estimating the baseline risk of G2-LRB using CNN-based automatic plan generation. The present results will contribute to the development of decision support tools aimed at identifying priority cases for pre-RT interventions, such as hydrogel spacer implantation.

We observed a high goodness-of-fit (*R*^2^ = 0.80, Fig. 4) and a small %MAE (1.24%, Table 1) in the G2-LRB-LKB-NTCP prediction. Although direct comparisons are challenging because of different metrics, our results were superior or at least similar to the findings of a previous study using RapidPlan^TM^ (a commercial KBP system based on the OVH method).^25^ They reported that the maximum differences between planned and predicted G2-LRB-NTCP were −1.9% for RapidArc plans and −3.1% for Tomotherapy plans. This difference may be explained by the complexity of the extracted features used in each model. U-net incorporates not only the geometric features used in the OVH method,^26^ but also considers mutual trade-offs among OARs.^14^^,^^15^ Notably, predicted dose distributions in the present study reflected the rectum-sparing patterns commonly observed in the treatment plans of prostate RT (Fig. 2A). Although automatic feature extraction in U-net offers advantages over traditional methods,^13^ Shiraishi and Moore^27^ achieved excellent results in dose predictions and rectal NTCP predictions for prostate cancer RT using a modified OVH method combined with artificial neural networks with only simple geometric features (eg, PTV volume, number of fields, distance from PTV) compared with those used in U-net. This finding suggests that such simple geometric features may still be important for predicting rectal DVH and NTCP. Therefore, further studies are warranted to identify which models more accurately and efficiently predict rectal NTCP using the same data set. Furthermore, our results also suggest that quality of life (QOL)-related outcomes such as stool frequency and sphincter control may be predictable before RT. Given that a recent phase 3 trial has shown the benefit of a hydrogel spacer in reducing bowel QOL decline,^4^ the prediction of expected QOL outcomes would also be helpful in decision support in RT for prostate cancer. However, it remains uncertain whether rectal DVH alone can account for these QOL-related outcomes because recent studies have reported an association between QOL-related outcomes and radiation doses to the pelvic floor muscles.^28^^,^^29^ Therefore, further research on the sites and mechanisms associated with QOL-related outcomes is needed to refine the estimation model.

As an overall measure of model performance, the mean iDSC_≥0Gy_ of our 2D U-net model was 0.87 for test cases (Fig. 3B), which was similar or slightly inferior to those in other studies using 2D U-net and patch-based 3D U-net.^10^^,^^14^ In line with other studies, a decline in DSCs was observed in low to intermediate isodose volumes (10-40 Gy), presumably because of variations in optimization methods among planners.^10^^,^^14^ Nevertheless, G2-LRB-NTCP was accurately predicted in the present study (Fig. 4, Table 1). This may be attributed to the high predictive performance above 50 Gy (mean iDSC_≥50Gy_ = 0.90, Fig. 3; *R*^2^ ≥ 0.73, Fig. 4) and the high seriality of the rectum, as indicated in Eq. (4) with a relatively low *n* of 0.19. Therefore, for future clinical applications, a model that effectively predicts an isodose volume > 50 Gy may sufficiently estimate the risk of G2-LRB. This appears to be biologically plausible based on early endoscopic evidence associating LRB with telangiectatic lesions localized to the anterior rectal wall receiving high doses (> 60 Gy).^30^ This also corresponds to previous clinical findings that rectum V_65Gy_–V_70Gy_ strongly correlated with LRB.^28^^,^^31^, ^32^, ^33^

Our U-net model also accurately predicted rectum V_50Gy_–V_70Gy_ within 2.00% of the prediction errors (Table 1), which was similar to those reported in other studies using deep learning models (< 4% errors).^12^, ^13^, ^14^ High predictability in rectal DVH may be because of nearly Pareto-optimal trade-offs between the PTV and rectum because planners are more likely to pay more attention to rectal high doses than to other OARs in the optimization process. A previous study demonstrated that training data sets comprising Pareto-optimal plans for 45 or more cases were sufficient to create a DVH prediction model with a clinically acceptable error for rectal NTCP.^34^ However, the robustness of the rectal NTCP prediction using our method remains unknown with our data from a single institution. Therefore, additional research is needed to clarify the effects of different delivery methods and planning goals.

The present study had several limitations. We used 2D U-net instead of 3D U-net because 2D U-net maintains model stability by treating slices as individual training data despite the small sample size (n = 60). However, the 2D U-net model is based on slice-by-slice predictions, and spatial information in the craniocaudal direction was lost (Fig. 2). Because a previous study demonstrated the superiority of 3D U-net to 2D U-net for dose predictions of breast cancer RT,^35^ there may be room for further improvements in predictive ability using the 3D model. Furthermore, our U-net model required contouring the PTV and OARs in addition to acquiring planning CT scan data for each new prediction, while the dose prediction itself takes several seconds per patient with the trained model. This was because the incorporation of ROI structures as a model input has been reported to enhance the accuracy of dose predictions more than using a CT scan alone.^12^ Moreover, similar to previous findings,^10^, ^11^, ^12^, ^13^, ^14^, ^15^, ^16^ our U-net model cannot generate a clinically deliverable treatment plan because it does not take into account physical constraints such as field geometry and leaf movements. Although this study focused on estimating rectal NTCP, the conversion of the predicted 3D dose distribution to a deliverable plan may be used to ensure that the predicted NTCP is at least an “achievable NTCP” for a particular irradiation method and that it may be used as is for irradiation.^36^ In addition, treatment plans for patients receiving hydrogel spacer implantation were not available because of the lack of insurance coverage before 2018 in our country. Recent studies demonstrated the feasibility of predicting the expected change in rectal DVH or NTCP after implantation^7^^,^^37^^,^^38^; however, these studies used the OVH method or manually optimized plans. Therefore, in future studies, we plan to perform a comparative study on the ability of traditional KBP methods (eg, OVH method) and U-net to predict rectal NTCP changes before and after hydrogel spacer implantation.

In addition to these limitations, several issues need to be addressed before clinical implementation. While our U-net model showed high goodness-of-fit for most NTCP metrics, the range of predicted probabilities for G2-LRB differed between the LKB and RS models (8.1%-18.6% for LKB; 0.3%-2.3% for RS; Fig. E3). This discrepancy is likely because of differences in toxicity grading systems and the use of older irradiation methods in the RS model. Therefore, institution-specific calibration between predicted NTCP values and observed toxicity rates may be desirable to achieve a more accurate prediction of rectal toxicity across different institutions, although externally validated NTCP models can provide relative risk assessments without calibration.^24^ Such calibration could support consistent decision-making based on a predefined cut-off for NTCP. Another limitation is the need to evaluate the clinical utility of our U-net model in decision-making for hydrogel spacer implantation. Although our approach is relatively straightforward, the effort required to train the model must be justified by its clinical utility. However, commonly used metrics, such as the area under the receiver operating characteristic curve values, may not adequately assess the predictive performance of the model in terms of decision-making. Instead, decision curve analysis can assess the net reduction in unnecessary interventions, such as hydrogel spacer implantation, under different harm-benefit ratios.^39^ This analysis would help determine whether our approach offers advantages over strategies where hydrogel spacers are employed in all patients or not employed in any patients.

## Conclusions

We demonstrated the feasibility of our CNN-based automatic plan generation model to estimate the baseline risk of G2-LRB in prostate cancer VMAT. Although our CNN-based automatic plan generation model has clinical potential in the decision-making process for hydrogel spacer implantation, several issues need to be addressed before clinical implementation. Future studies should focus on validating the reproducibility of the model across different irradiation techniques and planning goals, developing institution-specific calibration of NTCP values to improve predictive accuracy in the clinical setting, and evaluating the clinical utility of the model using statistical approaches such as decision curve analysis. These efforts would contribute to the development of a standardized decision-making approach for hydrogel spacer implantation.

## Disclosures

Nagoya City University filed a patent application with the Japan Patent Office on April 11, 2024, listing Seiya Takano and Natsuo Tomita as inventors (patent application number, 2024-64305). The other authors have nothing to disclose.

## Appendix Supplementary materials

Revised_supplementary_materials_ARO_clean.pdf Image, application 1

## Footnotes

Supplementary material associated with this article can be found in the online version at doi:10.1016/j.adro.2025.101739.
