# Supplementary material for: Risk Estimation of Late Rectal Toxicity Using a Convolutional Neural Network-based Dose Prediction in Prostate Cancer Radiation Therapy
Source: Adv Radiat Oncol. 2025 Feb 15;10(4):101739. doi: 10.1016/j.adro.2025.101739 (PMC11950957; doi:10.1016/j.adro.2025.101739)
Supplement: Revised_supplementary_materials_ARO_clean.pdf [file mmc1.pdf]

Supplementary materials

Table E1 Planning goals for targets and organs at risk

**Structure**		**Constraint**
**PTV**		
	$D_{50\%}$ (Gy)	= 74.8 Gy (prescribed dose)
	$D_{95\%}$ (Gy)	≥95% of the prescribed dose
	D_{max} (Gy)	≤110% of the prescribed dose
	D_{mean} (Gy)	≥99% and ≤103% of the prescribed dose
	$V_{67.3Gy}$ (%)	≥96%
**Rectum**		
	$V_{75.1Gy}$ (%)	≤0%
	$V_{57.7Gy}$ (%)	≤18%
	$V_{38.5Gy}$ (%)	≤35%
**Bladder**		
	$V_{75.1Gy}$ (%)	≤0%
	$V_{62.5Gy}$ (%)	≤25%
	$V_{38.5Gy}$ (%)	≤50%
**Bowel**		
	D_{max} (Gy)	≤65 Gy
**Femoral head**		
	D_{max} (Gy)	≤37.4 Gy

Abbreviations: PTV = planning target volume.

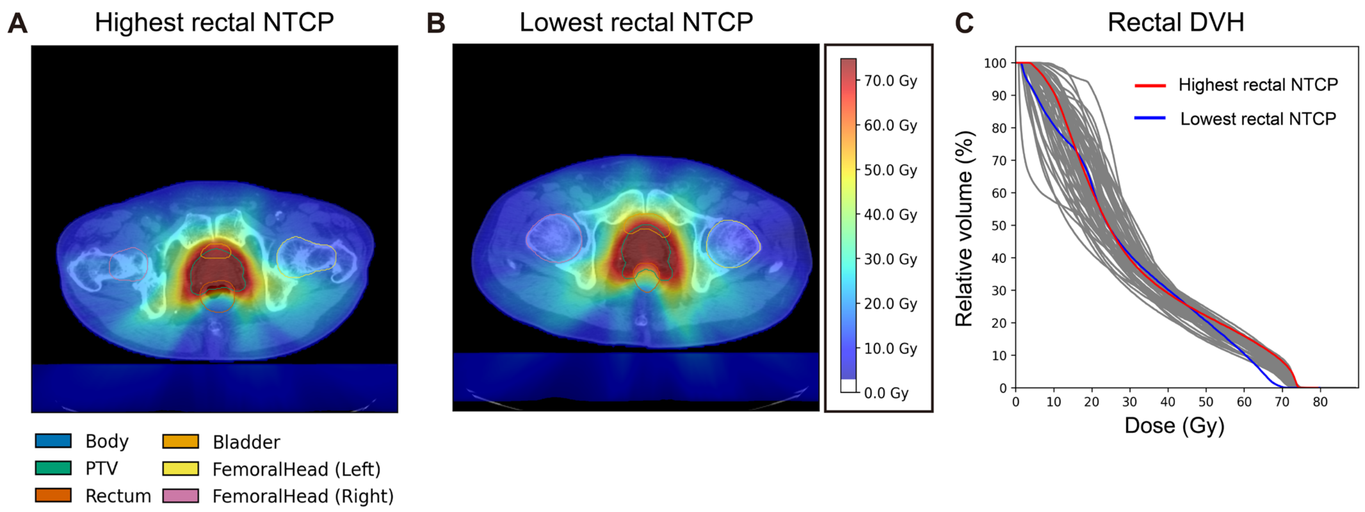

Fig. E1. Representative dose distributions of the training cases with the (A) highest and (B) lowest G2-LRB-LKB-NTCP values. (C) Rectal DVH curves of all training cases (n = 60). The rectal DVH curves with the highest (15.01%) and lowest G2-LRB-LKB-NTCP values (9.69%) are highlighted by the red and blue lines, respectively.

Abbreviations: G2 = grade ≥2; LRB = late rectal bleeding; LKB = Lyman-Kutcher-Burman; NTCP = normal tissue complication probability; DVH = dose-volume histogram.

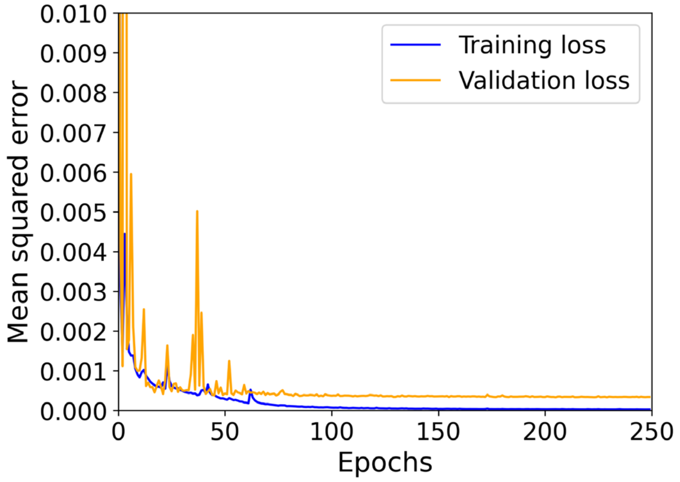

Fig. E2. Training and validation loss curves from one of the folds.

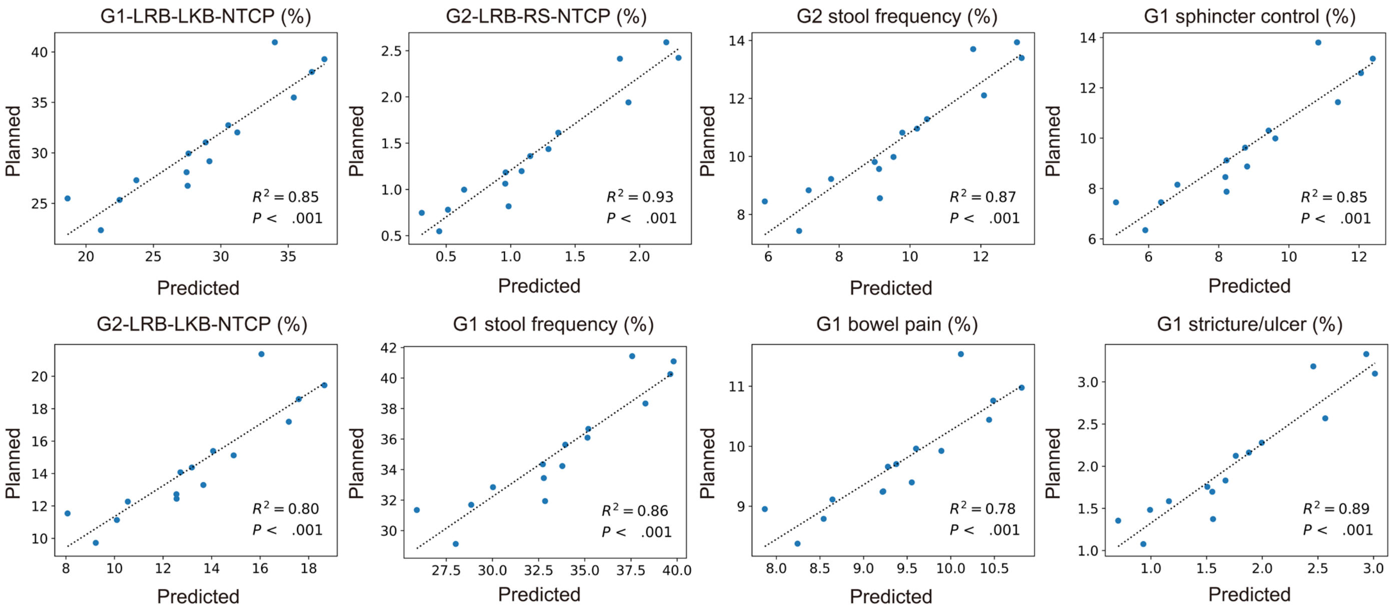

Fig. E3. Scatter plots of the linear regression analysis between planned and predicted NTCP values with different models (LKB and RS models) and other fitting parameters for multiple toxicity endpoints.

Abbreviations: NTCP = normal tissue complication probability; LKB = Lyman-Kutcher-Burman; RS = relative seriality; G1 = grade ≥1; LRB = late rectal bleeding; G2 = grade ≥2.
